# A missense variant at the RAC1-PAK1 binding site of *RAC1* inactivates downstream signaling in VACTERL association

**DOI:** 10.1038/s41598-023-36381-0

**Published:** 2023-06-16

**Authors:** Rie Seyama, Masashi Nishikawa, Yuri Uchiyama, Keisuke Hamada, Yuka Yamamoto, Masahiro Takeda, Takanori Ochi, Monami Kishi, Toshifumi Suzuki, Kohei Hamanaka, Atsushi Fujita, Naomi Tsuchida, Eriko Koshimizu, Kazuharu Misawa, Satoko Miyatake, Takeshi Mizuguchi, Shintaro Makino, Takashi Yao, Hidenori Ito, Atsuo Itakura, Kazuhiro Ogata, Koh-ichi Nagata, Naomichi Matsumoto

**Affiliations:** 1 Department of Human Genetics, Yokohama City University Graduate School of Medicine, Fukuura 3-9, Kanazawa-ku, Yokohama, 236-0004 Japan; 2 Department of Obstetrics and Gynecology, Juntendo University Faculty of Medicine, Tokyo, Japan; 3 Department of Molecular Neurobiology, Institute for Developmental Research, Aichi Developmental Disability Center, 713-8 Kamiya, Kasugai, Aichi 480-0392 Japan; 4 Department of Biological Sciences, Nagoya University, Nagoya, Japan; 5 Department of Rare Disease Genomics, Yokohama City University Hospital, Yokohama, Japan; 6 Department of Biochemistry, Yokohama City University Graduate School of Medicine, Yokohama, Japan; 7 Department of Pediatric Surgery, Juntendo University School of Medicine, Tokyo, Japan; 8 Department of Human Pathology, Juntendo University School of Medicine, Tokyo, Japan; 9 Department of Obstetrics and Gynecology, Keiai Hospital, Saitama, Japan; 10 RIKEN Center for Advanced Intelligence Project, Tokyo, Japan; 11 Department of Clinical Genetics, Yokohama City University Hospital, Yokohama, Japan; 12 Department of Obstetrics and Gynecology, Juntendo University Urayasu Hospital, Urayasu, Japan; 13 Department of Human Pathology, Juntendo University Graduate School of Medicine, Tokyo, Japan; 14 Department of Neurochemistry, Nagoya University Graduate School of Medicine, 65 Tsurumai-cho, Nagoya, Japan 466-8550

**Keywords:** Genetics, Clinical genetics, Gene regulation, Sequencing

## Abstract

*RAC1* at 7p22.1 encodes a RAC family small GTPase that regulates actin cytoskeleton organization and intracellular signaling pathways. Pathogenic *RAC1* variants result in developmental delay and multiple anomalies. Here, exome sequencing identified a rare de novo* RAC1* variant [NM_018890.4:c.118T > C p.(Tyr40His)] in a male patient. Fetal ultrasonography indicated the patient to have multiple anomalies, including persistent left superior vena cava, total anomalous pulmonary venous return, esophageal atresia, scoliosis, and right-hand polydactyly. After birth, craniofacial dysmorphism and esophagobronchial fistula were confirmed and VACTERL association was suspected. One day after birth, the patient died of respiratory failure caused by tracheal aplasia type III. The molecular mechanisms of pathogenic *RAC1* variants remain largely unclear; therefore, we biochemically examined the pathophysiological significance of RAC1-p.Tyr40His by focusing on the best characterized downstream effector of RAC1, PAK1, which activates Hedgehog signaling. RAC1-p.Tyr40His interacted minimally with PAK1, and did not enable PAK1 activation. Variants in the *RAC1* Switch II region consistently activate downstream signals, whereas the p.Tyr40His variant at the RAC1-PAK1 binding site and adjacent to the Switch I region may deactivate the signals. It is important to accumulate data from individuals with different *RAC1* variants to gain a full understanding of their varied clinical presentations.

## Introduction

The RAC subfamily of small guanosine triphosphate(GTP)ases consists of RAC1, RAC2 and RAC3, which share approximately 90% amino acid identity. *RAC1* is ubiquitously expressed and regulates intracellular signaling pathways and cytoskeletal dynamics, which influence cell adhesion, morphology, migration, and cell cycle progression in various cell types^1,2^. *Rac1* knockout mice were embryonic lethal due to the failure in formation of the three germ layers during gastrulation resulting in congenital heart defects^3,4^. As a small GTPase, RAC1 cycles between active/GTP-bound states and inactive/guanosine diphosphate(GDP)-bound states via conformational changes mainly in the Switch I (amino acids 25–39) and Switch II (amino acids 57–75) regions^5^. The GTP/GDP-bound states are controlled by various guanine nucleotide exchange factors (GEFs) and GTPase-activating proteins (GAPs). While GEFs facilitate GDP release and GTP loading to activate GTPases, GAPs enhance intrinsic GTP-hydrolysis to inactivate GTPases^6^. The GTP-bound small GTPases specifically interact with and activate downstream effector molecules in spatiotemporally regulated manners.

GTP-bound RAC1 activates a large number of effector proteins, such as p21-activated kinase 1–3 (PAK1, PAK2, and PAK3)^7,8^. Of particular note, the RAC1-PAK1 pathway is involved in nuclear translocation of the transcription factor, Gli, which is indispensable for activation of Hedgehog (Hh) signaling^9^. Hh signaling is a key regulator of intercellular communication during the metazoan development^10,11^. Notably, Hh signaling defects have been considered to be related to VACTERL association (vertebral anomalies, anal atresia, cardiac malformations, trachesophageal fistula, renal anomalies, and limb anomalies) since the perturbation of this signaling and its downstream pathway in mice phenocopies many of these human deformities while no variants in this pathway genes have been found in relation to VACTERL association in humans^12^.

In this study, we describe a case with VACTERL association and a novel de novo variant of *RAC1* [NM_018890.4:c.118T > C p.(Tyr40His)] at the RAC1-PAK1 binding site, which is adjacent to the Switch I region. From the previous study, *RAC1* missense variants in the Switch II region can change GTP/GDP-bound states and cause the constitutive activity of downstream pathways including PAK family kinases^13,14^. However, effects of other *RAC1* missense variants are still undetermined. We conducted in vitro functional analyses to understand the pathogenic effects of this novel *RAC1* variant.

## Results

### Clinical features

**Figure 1:**
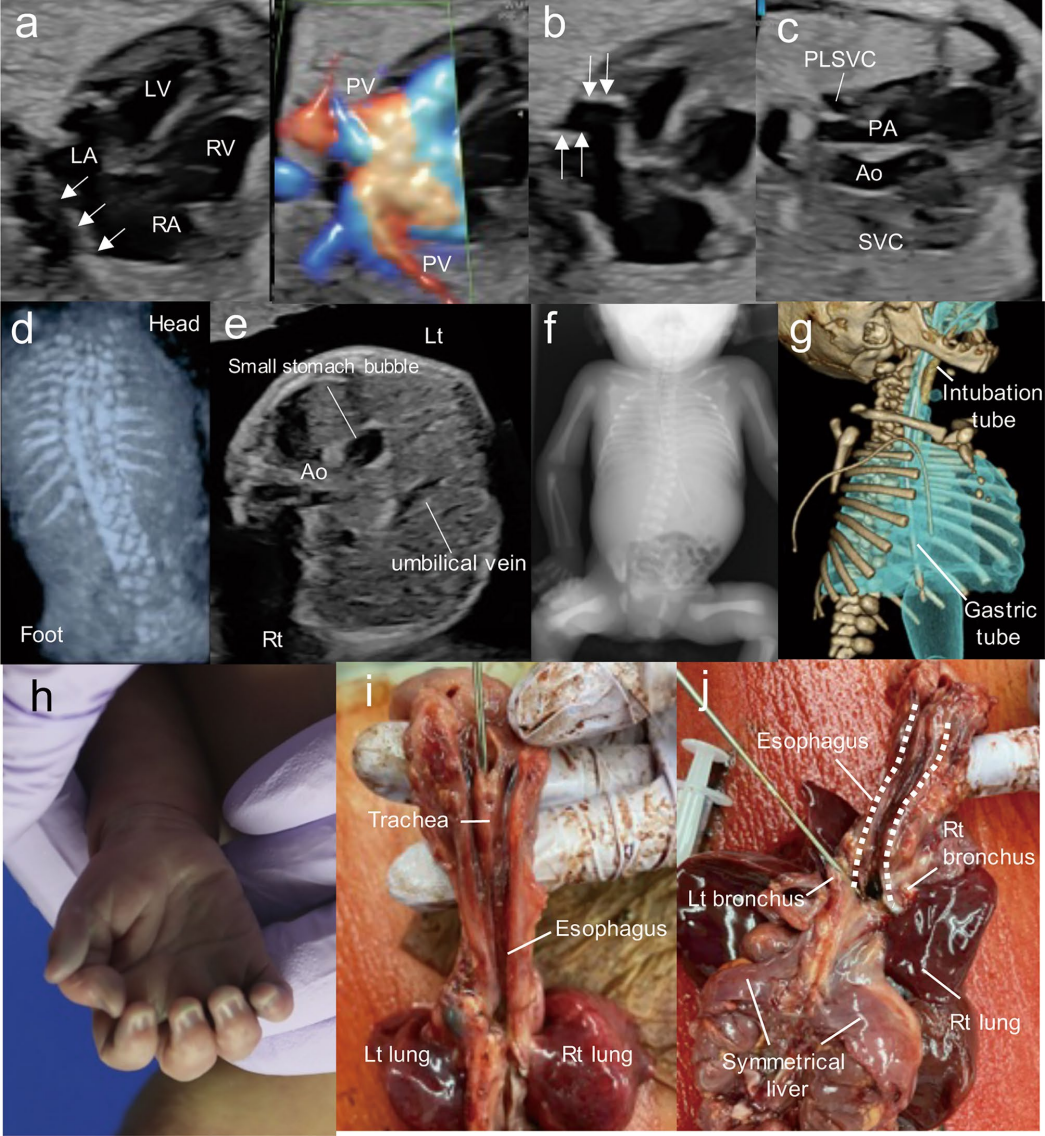
Clinical features of a case with a *RAC1* variant. (**a**–**e**) Fetal ultrasonography of the proband at 28 weeks of gestation. (**a**) Posterior venous confluence (white arrows) was recognized on a 4-chamber view of isolated total anomalous pulmonary venous return (TAPVR). (**b**) Enlarged coronary sinus (white arrows) implied persistent left superior vena cava (PLSVC). (**c**) Abnormal three vessel view showed the PLSVC. (**d**) Fetal spine 3D ultrasonography with skeleton mode indicated scoliosis. (**e**) Small stomach bubble implied esophageal atresia on axial view, which was a finding associated with tracheal agenesis type III. (**f**) Scoliosis was recognized at birth by X-ray imaging. (**g**) 3D computed tomography imaging showed that an intubation tube was not located at the bifurcation of the trachea, indicating tracheal agenesis type III. (**h**–**j**) Photographs of the patient at autopsy. He had right-hand polydactyly (**h**). The trachea was blind-ended (**i**) and the esophagus was connected to the right lung, indicating tracheal agenesis type III and esophagotracheal fistula (**j**), respectively. Symmetrical liver was present (**j**). *Ao* aorta, *LA* left atrium, *Lt* left, *LV* left ventricle, *RA* right atrium, *Rt* right, *RV* right ventricle, *PV* pulmonary vein, *PLSVC* persistent left superior vena cava.

Detailed clinical features of our patient are shown in Table 1 and Fig. 1. The proband (II-3) was born from his mother’s third pregnancy. Fetal ultrasonography at 28 weeks of gestation identified multiple congenital anomalies: total anomalous pulmonary venous return (Fig. 1a), enlarged coronary sinus (Fig. 1b) and a fourth vessel in the three vessel and trachea view (Fig. 1c) implying persistent left superior vena cava, scoliosis (Fig. 1d), and right-hand polydactyly. A small stomach bubble (Fig. 1e) with polyhydramnios implied esophageal atresia. The fetal karyotype by amniocentesis was normal (46,XY). An emergency cesarean section was performed at 30 weeks and 3 days of gestation because of non-reassuring fetal status. His birth weight was 1406 g (− 0.69 SD) and Apgar scores were 3 (pulse: 2, activity: 1, others: 0) at 1 and 5 min after birth, respectively.

**Table 1:** Clinical features of the current patient and a previously reported patient with *RAC1* variants adjacent to the Switch I region.

	Our case	Individual 2^a^
Clinical information		
Gender	Male	Male
Race	Japanese	European
Age at examination	30 weeks and 3 days of gestation	9 years
Height	40 cm (0.10 SD)	128 cm (− 2.5 SD)
Weight	1406 g (− 0.69 SD)	24 kg (− 0.5 SD)
Mutation (GenBank: NM_018890.4)		
Chromosome position (GRCh37/hg19)	chr7:6431565	chr7:6431563
gDNA change	c.118T > C	c.116A > G
Amino acid change	p.(Tyr40His)	p.(Asn39Ser)
SIFT	0	0
PolyPhen-2	0.731	0.999
CADD	26.7	24.2
MutationTaster	Disease-causing	Disease-causing
ACMG/AMP Guidelines (Criteria)	Likely pathogenic (PS2, PM1, PM2, PP3)	Pathogenic (PS3, PM1, PM2, PM6, PP3, PP4)
Developmental and neurological findings		
Intellectual disability (degree)	NA	Yes (mild-moderate)
Epilepsy	NA	No
Hypotonia	NA	No
Brain MRI abnormalities		
	Not performed	Cerebellar abnormalities, Hypoplasia corpus callosum, Enlarged lateral ventricles, Enlarged fourth ventricle, Thin pons, Mega cisterna magna
Craniofacial dysmorphisms		
Low set ears	Yes	Yes
Micrognathism	Yes	Yes
Arched eyebrows	Yes	Yes
Prominent nasal bridge	No	No
Congenital abnormalities		
Cardiac abnormalities	PLSVC, TAPVR	No
Tracheoesophageal malformation	Esophagobronchial fistula	No
Skeletal abnormalities	Scoliosis, 10th thoracic vertebra hypoplasia; synostosis of 6th and 7th costa, tracheal aplasia	No
Hand deformities	Polydactyly 1st digit on right-hand	No
Other		
Neonatal feeding difficulties	Yes	Yes
Other	Died on the first day after delivery Polyhydramnios	Recurrent pneumonia Eczema

*NA* not available, *PLSVC* persistent left superior vena cava, *SD* standard deviation, *TAPVR*: total anomalous pulmonary venous return. The pathogenicity of each prediction score was judged according to the following values: SIFT < 0.05 (Deleterious), PolyPhen-2 0.65–1.0 (Damaging), and CADD (phred score) ≥ 20 (Pathogenic). ^a^Reijnders et al.^13^.

Multiple anomalies were confirmed at birth, including scoliosis (Fig. 1f), tracheal agenesis type III (Fig. 1g), low set ears, micrognathia, and right-hand polydactyly (Fig. 1h). He died of respiratory failure caused by tracheal agenesis type III (Fig. 1i,j) on the first day after delivery. Prenatally suspected esophageal atresia was determined to be esophagotracheal fistula at autopsy (Fig. 1i). His autopsy additionally revealed that he had 10th thoracic vertebra hypoplasia, 6th and 7th rib fusion, and symmetrical liver (Supplementary Fig. S1a). Other autopsy findings are presented in Supplementary Fig. 1. These clinical features were compatible with VACTERL association^15^.

### Identification of a pathogenic *RAC1* variant

**Figure 2:**
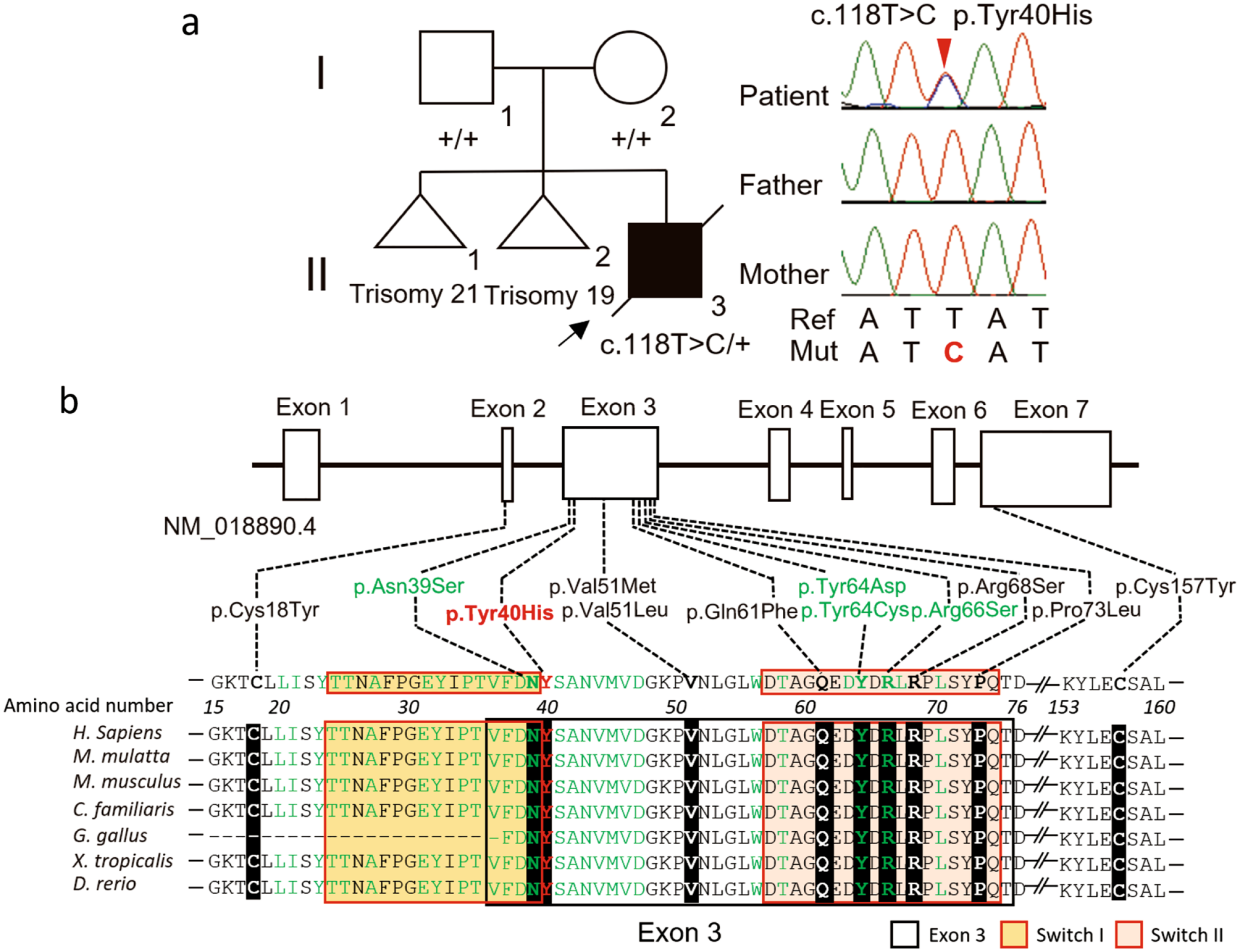
Pathogenic variants associated with RAC1-related neurodevelopmental disorder. (**a**) Family pedigree (left) and electropherograms showing the pathogenic variant in the family (right). Squares, a circle, and triangles indicate male, female, and miscarriages, respectively. Black arrow indicates the proband. Red arrow indicates the pathogenic variant. Ref, Mut, and + indicate reference, mutant, and normal alleles, respectively. (**b**) Schematic presentation of *RAC1*, with the coding exons shown as boxes and the introns as black lines. Dotted lines indicate the location of variants with their details shown below. Evolutionary conservation of amino acids subject to pathogenic missense variant from *D. rerio* to *H. sapiens* is shown. Red text indicates the proband’s variant. Green text indicates the location of the RAC1-PAK1 connecting regions (amino acids 20, 21, 23–25, 27, 31, 33, 34, 36–47, 56, 58, 63, 64, 66, 67, and 70)^16^. Bold characters indicate the location of previously reported *RAC1* pathogenic variants. White, yellow, and bright orange boxes indicate exon 3, Switch I region (amino acids 25–39), and Switch II region (amino acids 57–75), respectively.

Exome sequencing identified a missense variant in *RAC1* [NM_018890.4:c.118T > C p.(Tyr40His)] in the proband (II-3). Trio-based Sanger sequencing confirmed that this variant occurred de novo (it was not found in the biological parents, which was confirmed with microsatellite markers) (Fig. 2a) and was absent from our in-house exome database of 575 Japanese individuals and other control population databases. This variant is in exon 3 of *RAC1*, which encodes the RAC1-PAK1 binding site^16^ and part (4 amino acids) of the Switch I region (Fig. 2b). Many other pathogenic variants are clustered in exon 3 and Tyr40 is highly evolutionarily conserved among vertebrates (Fig. 2b). Consistently, this variant was judged as disease-causing by SIFT, PolyPhen-2, MutationTaster, and CADD (Table 1). This variant was also classified as a likely pathogenic variant based on the American College of Medical Genetics and Genomics (ACMG) and the Association for Molecular Pathology (AMP) guidelines [PS2 (de novo), PM1 (located in hot spot), PM2 (absent from control), and PP3 (computational evidence)]^17^.

### Biochemical properties of RAC1-p.Tyr40His

**Figure 3:**
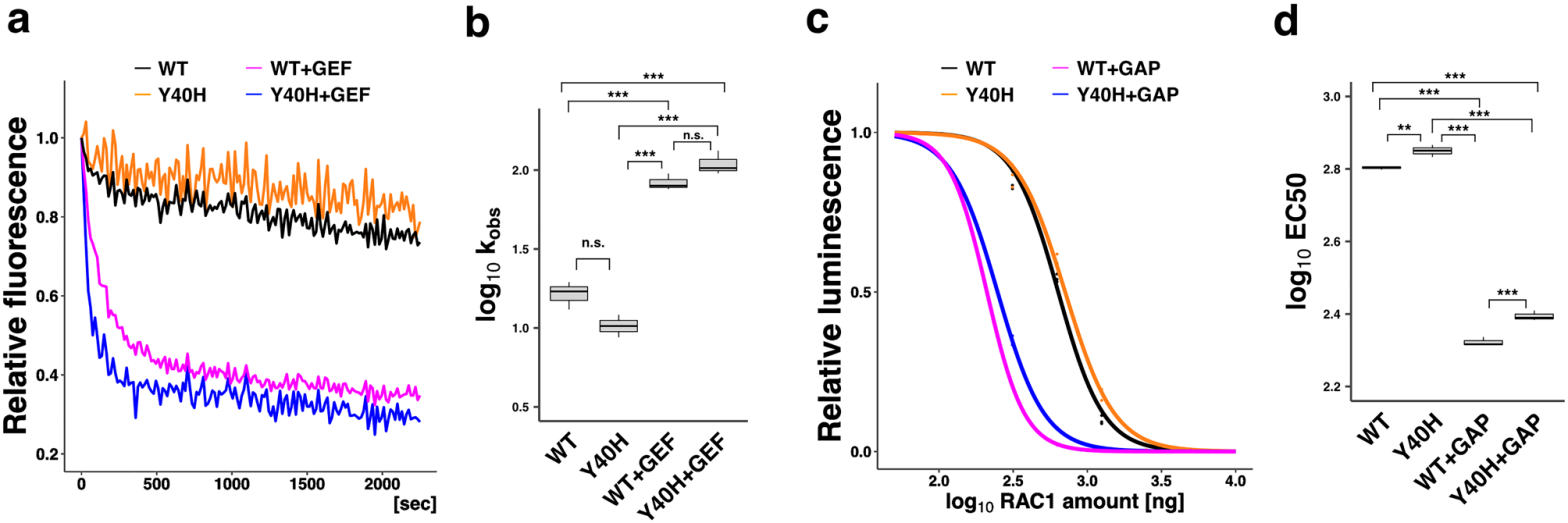
Effects of the p.Tyr40His variant on guanosine triphosphate (GTP)/guanosine diphosphate(GDP)-exchange and GTP-hydrolysis activities of RAC1. (**a**) Measurement of guanosine triphosphate (GTP)/guanosine diphosphate(GDP)-exchange activity. Recombinant His-tag-fused RAC1 (WT) or RAC1-p.Tyr40His (Y40H) was preloaded with fluorescent mantGDP, incubated with a non-hydrolysable GTP-analog in the presence or absence of His-tagged Trio-D1 (GEF), and relative fluorescence was monitored. (**b**) mantGDP-dissociation rates of WT and RAC1-p.Tyr40His were calculated as observed rate constants (k_obs_ [× 10^–5^ s^−1^]) from the results in (**a**). Number of replicates, N ≥ 4. Asterisks indicate statistically significant differences as determined by Tukey’s test. p.Tyr40His vs. WT, *p* = 0.0938; WT + GEF vs. WT, *p* < 0.001; p.Tyr40His + GEF vs. WT, *p* < 0.001; WT + GEF vs. p.Tyr40His, *p* < 0.001; p.Tyr40His + GEF vs. p.Tyr40His, *p* < 0.001; WT + GEF vs. p.Tyr40His + GEF, *p* = 0.3203. Boxes with the same letter are not significantly different. (**c**) Measurement of GTP-hydrolysis activity. Intrinsic activity of His-RAC1 (WT) and His-RAC1-p.Tyr40His in the presence or absence of His-tagged α-Chimerin (GAP) was analyzed by directly measuring changes in GTP concentration using a GTPase-Glo assay kit. (**d**) EC50 (half maximal effective concentration) was estimated from the sigmoidal fitted curve in (**c**). Number of replicates, N ≥ 4. *p*-value was calculated as in (**b**). p.Tyr40His vs. WT, *p* = 0.00862; WT + GAP vs. WT, *p* < 0.001; p.Tyr40His + GAP vs. WT, *p* < 0.001; WT + GAP vs. p.Tyr40His, *p* < 0.001; p.Tyr40His + GAP vs. p.Tyr40His, *p* < 0.001; WT + GAP vs. p.Tyr40His + GAP, *p* < 0.001. ***p < 0.001; **p < 0.01; *n.s.* not significant.

To investigate the properties of RAC1-p.Tyr40His, we examined its activation status. GTP/GDP-exchange and GTP-hydrolysis activities of this variant were compared with those of wild-type RAC1. When log_10_ k_obs_ (observed rate constant) was measured, the exchange activity of RAC1-p.Tyr40His was comparable to that of wild-type RAC1 (Fig. 3a,b). Notably, the exchange reaction of the variant with Trio-D1 (a GEF) was promoted to a level similar to that of the wild type (Fig. 3a,b). However, GTP-hydrolysis activity of RAC1-p.Tyr40His was significantly decreased compared with that of wild-type RAC1 (Fig. 3c,d). Meanwhile, α-Chimerin (a GAP) efficiently increased the hydrolysis activity, similarly to the wild type (Fig. 3c,d). Overall, therefore, RAC1-p.Tyr40His demonstrated a slightly lower GTP-hydrolysis activity than wild-type RAC1, while GTP/GDP-exchange activity was similar to that of the wild type. We assume that while RAC1-p.Tyr40His on its own may be a moderately activated version in vitro, it interacts with GEFs and GAPs and cycles between GDP-bound inactive and GTP-bound active states.

### Interaction of RAC1-p.Tyr40His with PAK1, a downstream effector

**Figure 4:**
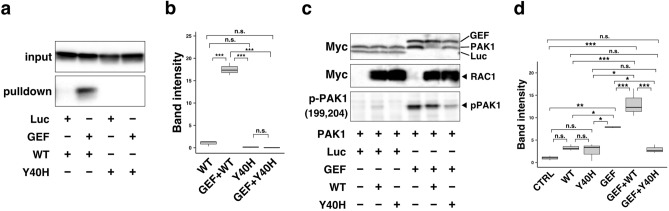
The in vitro interaction of RAC1 with PAK1, a downstream effector. (**a**) Interaction with the p21-binding domain (PBD) of PAK1. COS7 cells were transfected with pCAG-Myc-RAC1, -Myc-RAC1-p.Tyr40His, -Myc-Trio-D1 (GEF), and -Myc-Luciferase (Luc) vectors (0.3 μg each) in various combinations. Pull-down assays were conducted with GST-fused PAK1-PBD (5 μg each). Bound RAC1 proteins were detected by western blotting with anti-Myc. Total cell lysates were also immunoblotted with anti-Myc for normalization (input). (**b**) Relative band intensities of precipitated PAK1 were normalized against WT and are shown as fold-increase over the WT level below the image. Asterisks indicate statistically significant differences as determined by Tukey’s test. GEF + WT vs. WT, *p* < 0.001; p.Tyr40His vs. WT, *p* = 0.525; GEF + p.Tyr40His vs. WT, *p* = 0.454; p.Tyr40His vs. GEF + WT, *p* < 0.001; GEF + p.Tyr40His vs. GEF + WT, *p* < 0.001; GEF + p.Tyr40His vs. p.Tyr40His, *p* = 0.999. (**c**) Activation of exogenous PAK1. COS7 cells were transfected with pCAG-Myc-PAK1 and pCAG-Myc-Luciferase (Luc) or -Myc-Trio-D1 (0.1 μg each) together with pCAG-Myc vector (–), -Myc-RAC1, and -Myc-RAC1-p.Tyr40His (0.3 μg each). After 24 h, cell lysates were prepared and subjected to western blotting. Activated Myc-PAK1 was detected with an anti-phospho-Ser199/204-PAK1 antibody (bottom panel). Protein levels of Myc-Trio-D1, Myc-PAK1, Myc-Luc, and Myc-RAC1 were confirmed with an anti-Myc antibody (upper and middle panels). (**d**) Relative band intensities of phosphorylated PAK1 were normalized based on the PAK1 level and quantified against the positive control without RAC1 (lane 1) and are analyzed and shown as in (**b**). WT vs. CTRL, *p* = 0.51505; p.Tyr40His vs. CTRL, *p* = 0.81731; GEF vs. CTRL, *p* = 0.00187; GEF + WT vs. CTRL, *p* < 0.001; GEF + p.Tyr40His vs. CTRL, *p* = 0.66883; p.Tyr40His vs. WT, *p* = 0.99266; GEF vs. WT, *p* = 0.03513; GEF + WT vs. WT, *p* < 0.001; GEF + p.Tyr40His vs. WT, *p* = 0.99975; GEF vs. p.Tyr40His, *p* = 0.01383; GEF + WT vs. GEF, *p* < 0.001; GEF + p.Tyr40His vs. p.Tyr40His, *p* = 0.99970; GEF + WT vs. p.Tyr40His, *p* = 0.01623; GEF + p.Tyr40His vs. GEF, *p* = 0.02235; GEF + p.Tyr40His vs. GEF + WT, *p* < 0.001. (**b**,**d**) Number of replicates, N ≥ 3. *p*-value was calculated as in (Fig. 3b). ***p < 0.001; **p < 0.01; *p < 0.05; *n.s.* not significant.

Effects of the RAC1-p.Tyr40His variant on interaction with downstream signaling pathways were examined. We focused on PAK1, one of the most characterized RAC1 effector molecules, which is involved in actin cytoskeletal reorganization, cell adhesion and cell signaling^18^. We performed pull-down assays to evaluate the interaction of RAC1-p.Tyr40His with the PAK p21-binding domain (PAK1-PBD)^19^. RAC1-p.Tyr40His bound minimally with PAK1-PBD (Fig. 4a,b and Supplementary Fig. S2a–c). Given the biochemical analysis results (Fig. 3a–d), RAC1-p.Tyr40His on its own was presumed to be in a preferentially inactive GDP-bound state in COS7 cells (monkey kidney fibroblast-like cells). We then asked whether RAC1-p.Tyr40His interacts with PAK1 under the GTP-bound status. It is notable that RAC1-p.Tyr40His activated by Trio-D1 did not interact with PAK1-PBD under the conditions in which activated wild-type RAC1 efficiently interacted with PAK1-PBD (Fig. 4a,b and Supplementary Fig. S2a–c). Considering that GTP loading on the variant occurred normally in vitro (Fig. 3a,b), RAC1-p.Tyr40His was predicted to undergo GTP-dependent conformational change in response to Trio-D1 but it failed to form a complex with PAK1-PBD. We concluded that RAC1-p.Tyr40His did not activate PAK1 even in the biochemically “active” GTP-bound form.

We then tried to confirm the effects of the p.Tyr40His variant on PAK1 activity. To this end, RAC1-p.Tyr40His was expressed with Myc-PAK1 and Trio-D1 in COS7 cells, and PAK1 activation status was analyzed based on its autophosphorylation at Ser199 and Ser204^20^. Consequently, PAK1 was found to be minimally activated by RAC1-p.Tyr40His with Trio-D1, under the conditions where wild-type RAC1 activation caused PAK1 autophosphorylation (Fig. 4c,d, and Supplementary Fig. S2d–i). Trio-D1-mediated PAK1 activation without RAC1 may have occurred because of endogenous RAC proteins (Fig. 4c,d, and Supplementary Fig. S2g–i). These results indicate that RAC1-p.Tyr40His was not able to transmit upstream signaling to PAK1 regardless of GTP/GDP-binding state.

### Structural considerations for *RAC1* variants adjacent to the Switch I region

Based on the crystal structure of human RAC3 (which is highly homologous to RAC1) complexed with an effector, PAK1 (PDB: 2QME), we evaluated the structural effects of the p.Tyr40His variant and compared them with the previously reported human RAC1 p.Asn39Ser variant^13^. Asn39, Tyr40, and their interacting residues in RAC1 are fully conserved in RAC3.

**Figure 5:**
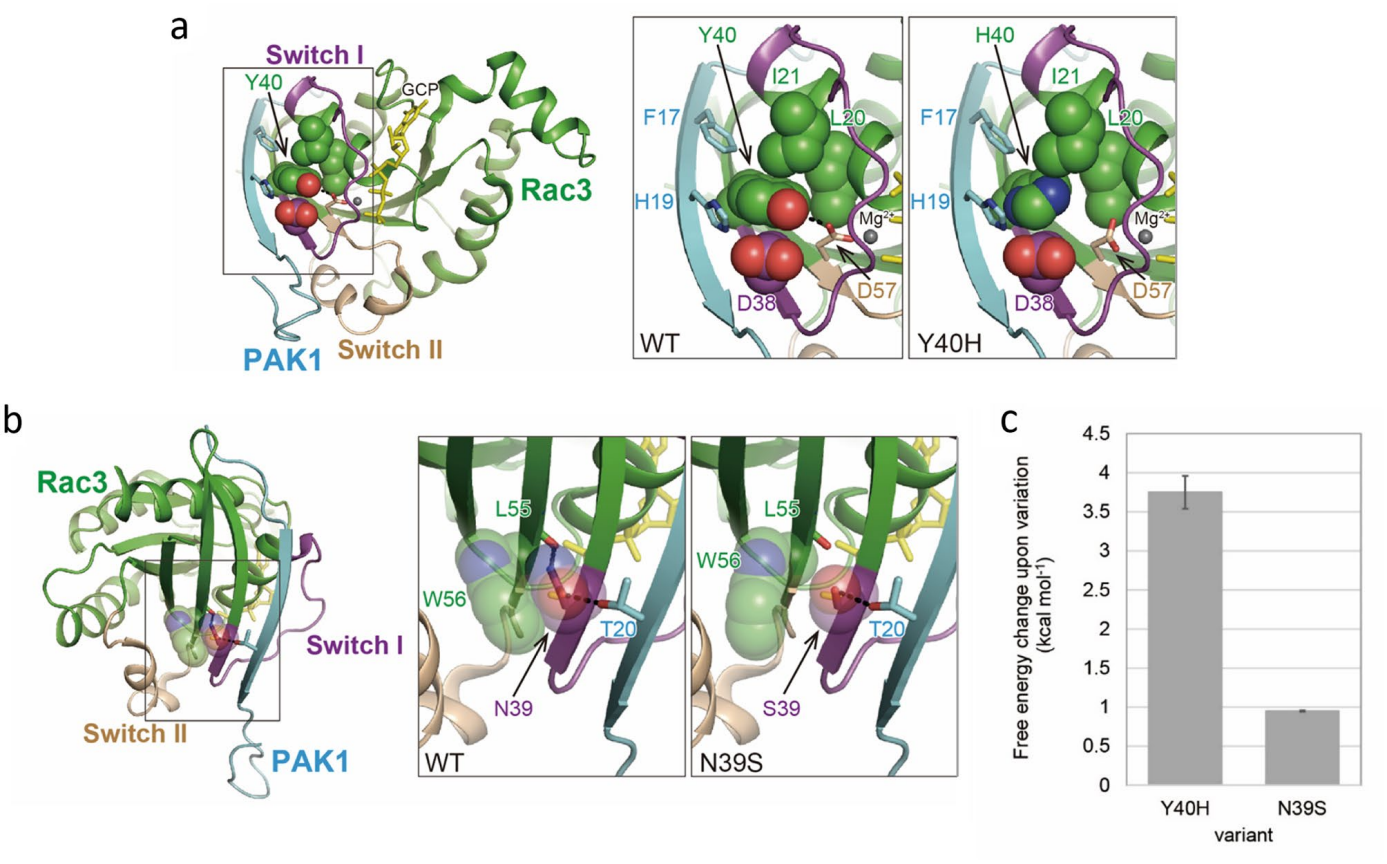
Structural considerations of the RAC1 variants. (**a**) Crystal structure of the human RAC3-PAK1 complex (PDB: 2QME) and close-up views of the region around Tyr40 for the wild type and the His40 variant model of RAC3. RAC3 and PAK1 in the complex are represented by green and cyan cartoons, respectively; the Switch I (Thr25–Asn39) and the Switch II (Asp57–Gln74) regions of RAC3 are shown in purple and beige, respectively. A non-hydrolyzable analog of GTP, phosphomethylphosphonic acid-guanylate ester (GCP), and the Mg^2+^ ion are shown as yellow sticks and a gray ball, respectively. (middle) Variant residue Tyr40 and the residues forming a hydrophobic core are shown as van der Waals spheres. Oxygen and nitrogen atoms are shown in red and blue, respectively. The dotted line represents a hydrogen bond (right). Structural model of the p.Tyr40His variant of RAC3 predicted by FoldX. (**b**) The complex structure from another angle and close-up views of the region around Asn39 of the wild type and the Ser39 variant model of RAC3 (middle and right). The variant residue Asn39, the modeled Ser39, and the residues forming hydrogen bonds with these residues are shown as sticks. Trp56, which makes van der Waals contacts with Asn39, is shown as a translucent sphere. Molecular structures were drawn using PyMOL Ver 2.5 (http://www.pymol.org). (**c**) The free energy changes for the indicated variants calculated by FoldX.

In this complex, Tyr40 of RAC3 is in the binding region for PAK1 and adjacent to the Switch I region (Thr25–Asn39) (Fig. 5a, left and middle). Tyr40 makes a hydrogen bond with Asp57. It also forms a hydrophobic core with Leu20, Ile21, and Asp38 (π orbitals) of RAC3, and Phe17 and His19 of PAK1, playing an important role in forming the RAC3-PAK1 complex (Fig. 5a, middle). Consequently, the p.Tyr40His variant would break the hydrogen bond and destabilize the hydrophobic core (Fig. 5a, right), resulting in impaired complex formation.

Asn39 also contributes to RAC3-PAK1 complex formation by making a network of hydrogen bonds with Thr20 of PAK1 and the main chain oxygen of Leu55 of RAC3 and also van der Waals contacts with Trp56 of RAC3 (Fig. 5b, left and middle). Based on the p.Asn39Ser variant model, Ser39 may maintain the hydrogen bond with Thr20 of PAK1 but could not interact with the main chain of Leu55 and Trp56 of RAC3 (Fig. 5b, right). This variant would therefore destabilize complex formation.

The value of the free energy change for each variation calculated by FoldX^21,22^ was significant for p.Tyr40His (3.75 ± 0.21 kcal/mol) and mild for p.Asn39Ser (0.95 ± 0.01 kcal/mol) (Fig. 5c), supporting our structural predictions.

## Discussion

In this study, a de novo* RAC1* variant [NM_018890.4:c.118T > C p.(Tyr40His)] located at the RAC1-PAK1 binding site, which is adjacent to the Switch I region, was identified in a Japanese patient. Compared with previously reported cases, this patient presented with very severe clinical features compatible with VACTERL association^15^, including cardiovascular anomalies, tracheoesophageal malformation, and skeletal anomalies, and died of respiratory failure on the first day after delivery at 30 weeks and 3 days of gestation.

All previously reported cases with *RAC1* variants located in Switch I or II regions involved altered GTP/GDP-bound states^13,14,23^. In the Online Mendelian Inheritance in Man (https://omim.org/), de novo* RAC1* variants have been found in intellectual developmental disorder (MIM#617751) with the highly variable phenotype, such as neurodevelopmental delay with abnormal brain magnetic resonance imaging findings, epilepsy, facial dysmorphisms, cardiovascular malformations and poor feeding. However, our case was more clinically severe with lethal malformations. Therefore, considering possible other variants, we checked the patient’s exome data but found no other deleterious variants (including single nucleotide variants and copy number variants).

To obtain insight into the pathophysiological mechanism of the p.Tyr40His variant, we performed in vitro characterization. Biochemical analyses revealed that this variant exhibited GTP/GDP-exchange activity comparable with that of wild-type RAC1 and GTP-hydrolysis activity only slightly affected. Therefore, the effect of this variant on biochemical properties did not seem to be significant. Given that the p.Tyr40His variant occurs at a residue adjacent to the Switch I region but not in the G-domain, which is essential for GTP/GDP-exchange and GTP-hydrolysis activities (Fig. 2b), it is plausible that this variant only slightly affected the biochemical properties of RAC1 (Fig. 3a–d). From these results, we consider that RAC1-p.Tyr40His preferentially binds GDP in the resting state and converts to the GTP-bound form in response to upstream signaling.

We next focused on the interaction of the p.Tyr40His variant with effector molecules. The etiology of VACTERL association is unclear, but abnormalities in Hh, fibroblast growth factor and NOTCH signaling are thought to be involved in its pathogenesis^24,25^. Recently, Tang et al. showed RAC1-PAK1 pathway-mediated Hh signaling via translocation of Gli into the nucleus followed by its transactivation using mouse embryonic fibroblasts^9^. We therefore selected PAK1 as a representative effector for RAC1. Notably, despite its apparently normal biochemical properties, RAC1-p.Tyr40His was neither able to interact with nor activate PAK1 even in the presence of a GEF (Fig. 3a–d, and Supplementary Fig. S3), when the variant is supposed to be an active GTP-bound form. We assume that this is because of the position of the p.Tyr40His variant; it is located in the effector-interaction region (amino acids 32–41) of RAC1, which overlaps with the Switch I region (amino acids 25–39) (Fig. 2b) and, therefore, affects affinity toward downstream effectors. Given that there are ~ 30 RAC1 effectors, the p.Tyr40His variant should show a specific spectrum of interactions with effectors, as is the case for RAC3^26,27^, leading to variant-specific clinical and pathological phenotypes. Other as yet unanalyzed downstream effector system(s) may also be hampered by the p.Tyr40His variation; however, it is possible that RAC1-p.Tyr40His normally activates other downstream effector(s).

It should be noted that the GTP-bound form of a small GTPase is generally recognized to be not only biochemically but also biologically activated and to activate downstream effectors. However, the results in Fig. 4 show that the GTP-bound state of the p.Tyr40His variant is not necessarily biologically active. We assume that RAC1-p.Tyr40His acts as a dominant-negative version for PAK1-mediated signaling, suggesting that the RAC-PAK1 signaling axis may partially underlie the pathophysiology of the p.Tyr40His variant (Supplementary Fig. S3). In this context, a variant of the neighboring residue, p.Asn39Ser, was also reported to be a dominant-negative version based on the lamellipodia formation assay using fibroblastic cells^13^. It should, however, be noted that, in addition to the PAK1 signaling, this variant is likely to disrupt multiple downstream pathways because a range of other RAC1 effectors are known to bind via their common CRIB domains with RAC1 (Supplementary Fig. S3)^28^. Further analyses are needed to uncover the precise pathophysiological mechanism of the p.Tyr40His variant.

The structural modeling studies also support our hypothesis that the p.Tyr40His variant inactivates RAC3-PAK1 signaling. Previous functional analyses of variants in the Switch II region showed constitutive activation of downstream signals in the RAC1-PAK1 axis^14^, and that variants adjacent to and in the Switch I region (p.Tyr40His and p.Asn39Ser) inactivate the signal. p.Tyr40His appeared to affect RAC3-PAK1 binding more strongly than p.Asn39Ser from the molecular modelling (Fig. 5c); therefore, it is reasonable that our p.Tyr40His case showed more severe phenotypes than the p.Asn39Ser case (Table 1).

In summary, we identified a novel *RAC1* variant in a patient with a severe and lethal phenotype. We showed that the variant, which is located at the RAC1-PAK1 binding site, may inactivate the downstream pathway. Further analyses of additional patients are needed to determine the effects of other pathogenic variants on other downstream genes that contribute to VACTERL association.

## Methods

### Subjects

The proband was born from the third pregnancy of healthy Japanese non-consanguineous parents. The previous two pregnancies resulted in miscarriages, each having chromosomal abnormalities: trisomy 21 in the first and trisomy 19 in the second. Written informed consent was obtained from the patient’s parents, in accordance with Japanese regulatory requirements. This study was approved by the Institutional Review Boards of Yokohama City University Faculty of Medicine under number A170525011 (modified B211100023) and Juntendo University Graduate School of Medicine (approval number 2017035). All experiments were performed in accordance with relevant guidelines and regulations. This practice was performed in accordance with the Declaration of Helsinki.

### Exome sequencing

Proband-based exome sequencing was performed for this family. Genomic DNA was extracted from umbilical cord blood leukocytes using QuickGene-610L (Fujifilm, Tokyo, Japan) according to the manufacturer’s protocol, captured using Twist Human Core Exome (Twist Bioscience, Kanagawa, Japan), and sequenced on an Illumina NovaSeq 6000 system (Illumina, San Diego, CA, USA) using 150 bp paired‐end reads. The exome data were processed as previously described^29^. The mean exome sequencing read depths in the RefSeq protein‐coding regions were from 76.6×. Briefly, reads were aligned to the GRCh37 human genome reference sequence (https://www.ncbi.nlm.nih.gov/assembly/GCF_000001405.13/) using NovoAlign (v3.02.13) (http://www.novocraft.com/products/novoalign/), and polymerase chain reaction (PCR) duplicates were excluded using Picard (https://broadinstitute.github.io/picard/). Indel realignment and recalibration of base‐quality scores were performed using Genome Analysis Tool Kit (GATK) (3.7‐0) (https://software.broadinstitute.org/gatk/). Called variants were annotated using ANNOVAR (http://annovar.openbioinformatics.org/en/latest/). Exonic and intronic variants within 30 bp from exon–intron boundaries were examined. Detected variants were filtered as follows. First, non‐pathogenic single nucleotide variants with minor allele frequencies > 1% in dbSNP137 (https://www.ncbi.nlm.nih.gov/projects/SNP/snp_summary.cgi?view+summary=view+summary&build_id=137), and variants found in more than five entries in our in‐house exome database, which includes data from 575 Japanese individuals, were excluded. Second, variants with minor allele frequencies > 1% in publicly available human variation databases were excluded. These were databases of the NHLBI Exome Sequencing Project (http://evs.gs.washington.edu/EVS/), the Human Genetic Variation Database (http://www.genome.med.kyoto-u.ac.jp/SnpDB/)^30^, the Exome Aggregation Consortium (http://exac.broadinstitute.org/), and the Tohoku Medical Megabank Organization (http://www.megabank.tohoku.ac.jp/english/). Third, candidate variants were selected based on variant type (nonsense, missense, frameshift, inframe, and splice site variant) using prediction software: SIFT (https://sift.bii.a-star.edu.sg/), PolyPhen‐2 (http://genetics.bwh.harvard.edu/pph2/), MutationTaster (http://www.mutationtaster.org/), CADD (https://cadd.gs.washington.edu/snv), ESEfinder (http://krainer01.cshl.edu/cgi-bin/tools/ESE3/esefinder.cgi), BDGP (https://www.fruitfly.org/seq_tools/splice.html), and SpliceAI^31^. We further shortlisted the remaining variants under the assumption of autosomal dominant, autosomal recessive, X‐linked dominant, and X‐linked recessive modes, and focused on rare variants in genes that are known to be related to neurodevelopmental disorders. In accordance with the ACMG/AMP guidelines^17^, candidate variants were classified as pathogenic, likely pathogenic, or variant of uncertain significance. Candidate variants and their familial cosegregation were confirmed by Sanger sequencing. Biological parentage was confirmed by assessing 12 microsatellite markers with Gene Mapper software v4.1.1 (Life Technologies Inc., Carlsbad, CA, USA). Primer sequences are available on request. Copy number variant analysis was performed as previously described^32^, but no pathogenic copy number variants were detected.

### Plasmids

Human *RAC1* and *PAK1* cDNAs were kind gifts from the late Prof. Alan Hall (University College, London, UK), and were subcloned into pCAG-Myc. The RAC1-p.Tyr40His variant was generated by site-directed mutagenesis using the KOD-Plus Mutagenesis kit (Toyobo Inc., Osaka, Japan) with pCAG-Myc-RAC1 as a template. Luciferase, as a control, was also subcloned into pCAG-Myc. RAC1 and RAC1-p.Tyr40His were also cloned into pTriEx-4 (Merck, Darmstadt, Germany). The p21-binding domain (PBD) in human PAK1 (amino acids 67–150) was amplified by PCR, and inserted into pGS21a (GenScript, Piscataway, NJ, USA). A Trio-D1 fragment (amino acids 1244–1969) containing the DH/PH domain of Trio, a Rac-GEF, and a Rac-GAP, α-Chimerin, were amplified by reverse transcription PCR of an embryonic day 16 mouse brain RNA pool and cloned into pCAG-Myc or pTriEx-4. All constructs were verified by Sanger sequencing.

### Antibodies

Anti-Myc and anti-phospho-PAK1 (Ser199/204) antibodies were purchased from Medical & Biological Laboratories (Nagoya, Japan, Cat# M047-3, RRID: AB_591112) and Cell Signaling Technology Japan (Tokyo, Cat# 2605, RRID:AB_2160222), respectively.

### GTP/GDP-exchange and GTP-hydrolysis assays

Preparation and purification of His-tag-fused RAC1, RAC1-p.Tyr40His, Trio-D1, and α-Chimerin were performed with Ni–NTA agarose (Qiagen Inc., Germantown, MD, USA) according to the manufacturer’s instructions^33^. To assess the basal GTP/GDP-exchange reactions, the release of methylanthraniloyl (mant)-GDP (Sigma-Aldrich, St Louis, MO, USA) was measured^33^. Intrinsic GTP-hydrolysis activity was assayed by monitoring changes in GTP concentration using a GTPase assay kit (GTPase-Glo Assay Kit, Promega, Madison, WI, USA)^34^.

### Cell culture and transfection

COS7 cells were cultured as previously described^26,35^. Transient transfection was performed using polyethyleneimine MAX reagent (Polysciences Inc., Warrington, PA, USA).

### Pull-down assays of GTP-bound RAC1 and RAC1-p.Tyr40His

Pull-down assays were performed as described previously^36^. A glutathione S-transferase (GST)-fused PBD of PAK1 was expressed in *Escherichia coli* BL21 (DE3) and purified using Glutathione Sepharose™ 4B (Cytiva, Marlborough, MA, USA) according to the manufacturer’s instructions^33^. Twenty-four hours after transfection with pCAG-Myc-RAC1, -RAC1-p.Tyr40His, -Luciferase (Luc), and -Trio-D1 (0.3 μg each/35 mm dish), either alone or in combination, COS7 cells were lysed with pull-down buffer (50 mM Tris–HCl, pH 7.5, 150 mM NaCl, 5 mM MgCl_2_, 0.1% SDS, 1% Nonidet P-40, and 0.5% deoxycholate). After insoluble materials were removed by centrifugation, the supernatant was incubated for 30 min at 4 °C with Glutathione Sepharose 4B beads (GE Healthcare Life Sciences, Buckinghamshire, England) to which GST-PBD of PAK1 was bound. Bound proteins were analyzed by western blotting using an LAS-4000 luminescent image analyzer (GE Healthcare Life Sciences).

### Statistical analysis

Statistical significance for multiple comparisons by Tukey’s test was set as *p* < 0.05 (https://cran.r-project.org/web/packages/multcomp/multcomp.pdf) using R (https://intro2r.com/citing-r.html; https://cran.r-project.org/doc/FAQ/R-FAQ.html#Citing-R).

### Structural considerations for the RAC1 variant

We referred to the crystal structure of human RAC3, which is highly homologous to RAC1, complexed with an effector, PAK1 (PDB: 2QME), to evaluate and compare the structural effects of the novel missense variant found in this study, RAC1-p.Tyr40His, with those of the known variant, p.Asn39Ser^13^. We used the program FoldX^21,22^ to calculate the free energy change resulting from the RAC1 variant. Molecular images were drawn using PyMOL Ver 2.5 (Schrödinger, LLC, New York, USA, http://www.pymol.org).

### Consent to participate

Informed consent was obtained from all participants included in the study.

## Supplementary Information

Supplementary Information.

## Data Availability

The data that support the findings of this study are available from the corresponding author upon reasonable request. All computational tools/codes and datasets used in this study can be downloaded through the following websites. ANNOVAR: http://annovar.openbioinformatics.org/en/latest/. BDGP: https://www.fruitfly.org/seq_tools/splice.html. CADD: https://cadd.gs.washington.edu/snv. Crystal structure of human RAC3 in complex with CRIB domain of human PAK1: https://www.rcsb.org/3d-view/2QME. dbSNP137: https://www.ncbi.nlm.nih.gov/projects/SNP/snp_summary.cgi?view+summary=view+summary&build_id=137. ESEfinder: http://krainer01.cshl.edu/cgi-bin/tools/ESE3/esefinder.cgi. The Exome Aggregation Consortium: http://exac.broadinstitute.org/. GRCh37 human genome reference sequence: https://www.ncbi.nlm.nih.gov/assembly/GCF_000001405.13/. Human Genetic Variation Database: http://www.genome.med.kyoto-u.ac.jp/SnpDB/. MutationTaster: http://www.mutationtaster.org/. NHLBI Exome Sequencing Project: http://evs.gs.washington.edu/EVS/. Picard: https://broadinstitute.github.io/picard/. PolyPhen‐2: http://genetics.bwh.harvard.edu/pph2/. R: https://intro2r.com/citing-r.html; https://cran.r-project.org/doc/FAQ/R-FAQ.html#Citing-R. SIFT: https://sift.bii.a-star.edu.sg/. The Tohoku Medical Megabank Organization: http://www.megabank.tohoku.ac.jp/english/.
