# Supplementary material for: A missense variant at the RAC1-PAK1 binding site of RAC1 inactivates downstream signaling in VACTERL association
Source: Sci Rep. 2023 Jun 16;13:9789. doi: 10.1038/s41598-023-36381-0 (PMC10275923; doi:10.1038/s41598-023-36381-0)
Supplement: Supplementary file 1 — Supplementary Information. [file 41598_2023_36381_MOESM1_ESM.docx]

Supporting Information

A missense variant at the RAC1-PAK1 binding site of *RAC1* inactivates downstream signaling in VACTERL association

Seyama R, et al.

**Contents**

**Supplemental Figure**

Fig. S1 The autopsy findings of our case 　　 p.2

Fig. S2 Original uncropped western blotting data of Fig. 4a and 4c p.3-5

Fig. S3 Scheme for the molecular mechanism of the p.Tyr40His variant 　　　 p.6

**Fig. S1 The autopsy findings of our case**

**
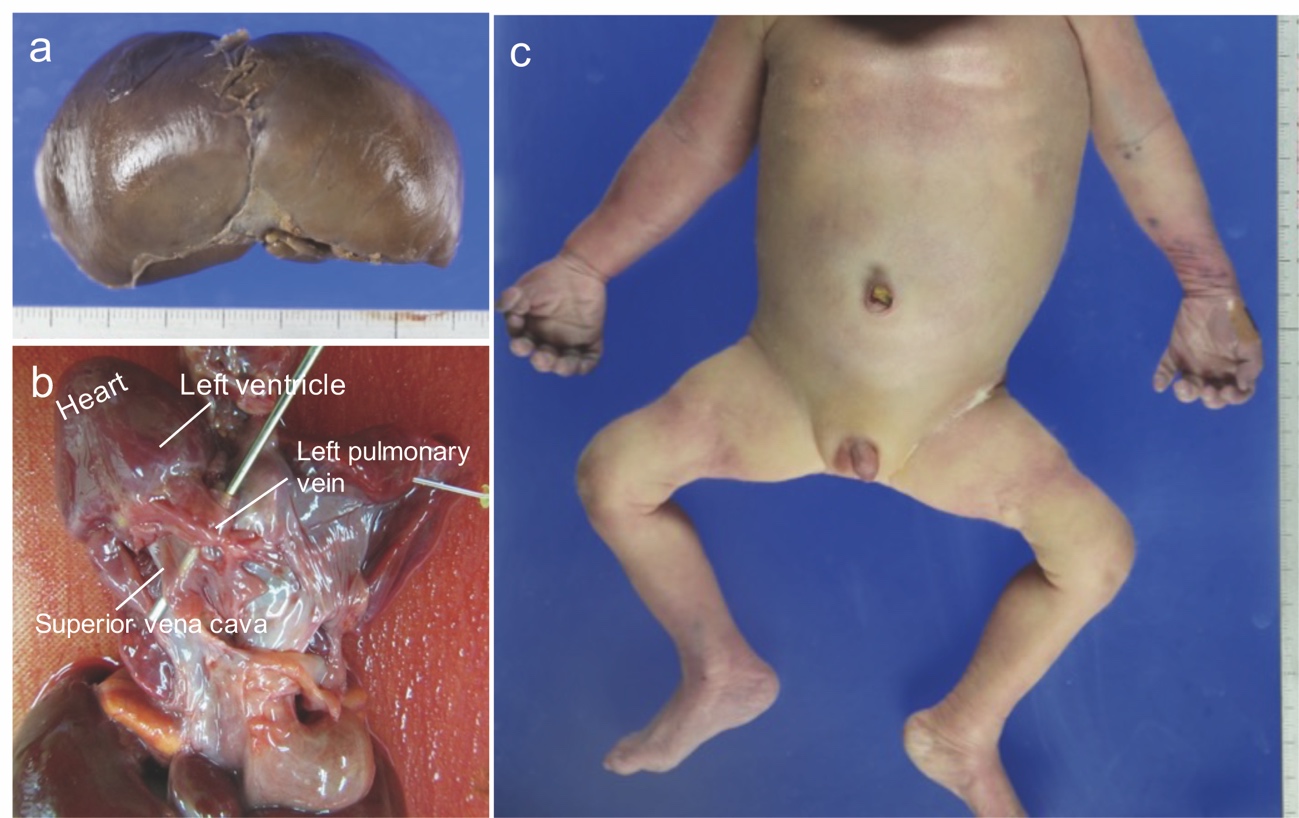
**

(a) Symmetrical liver. (b) Left pulmonary vein connected to superior vena cava. (c) Photograph of the case at autopsy.

**Fig. S2 Original uncropped western blotting data of Fig. 4a and 4c**

**
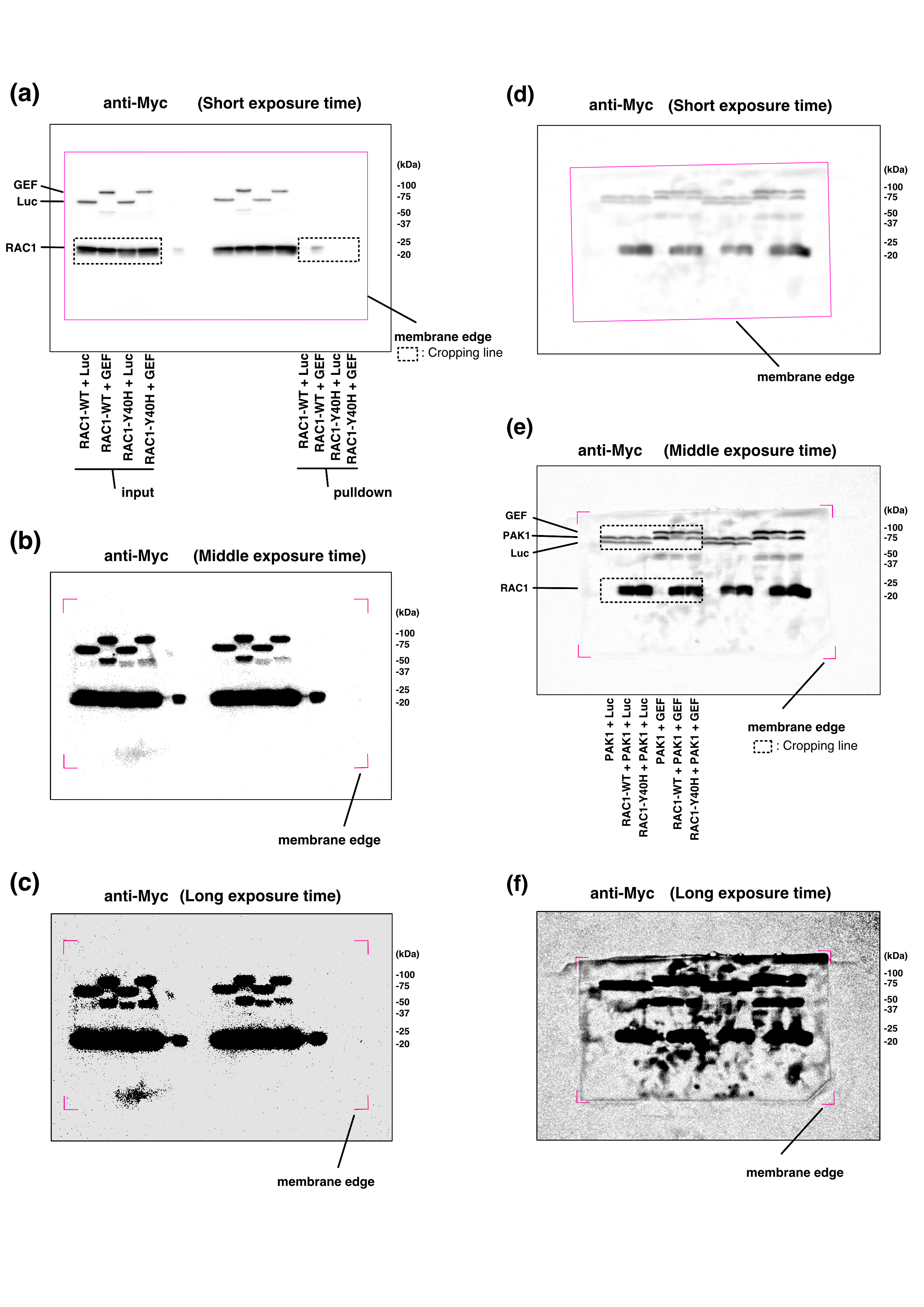
**


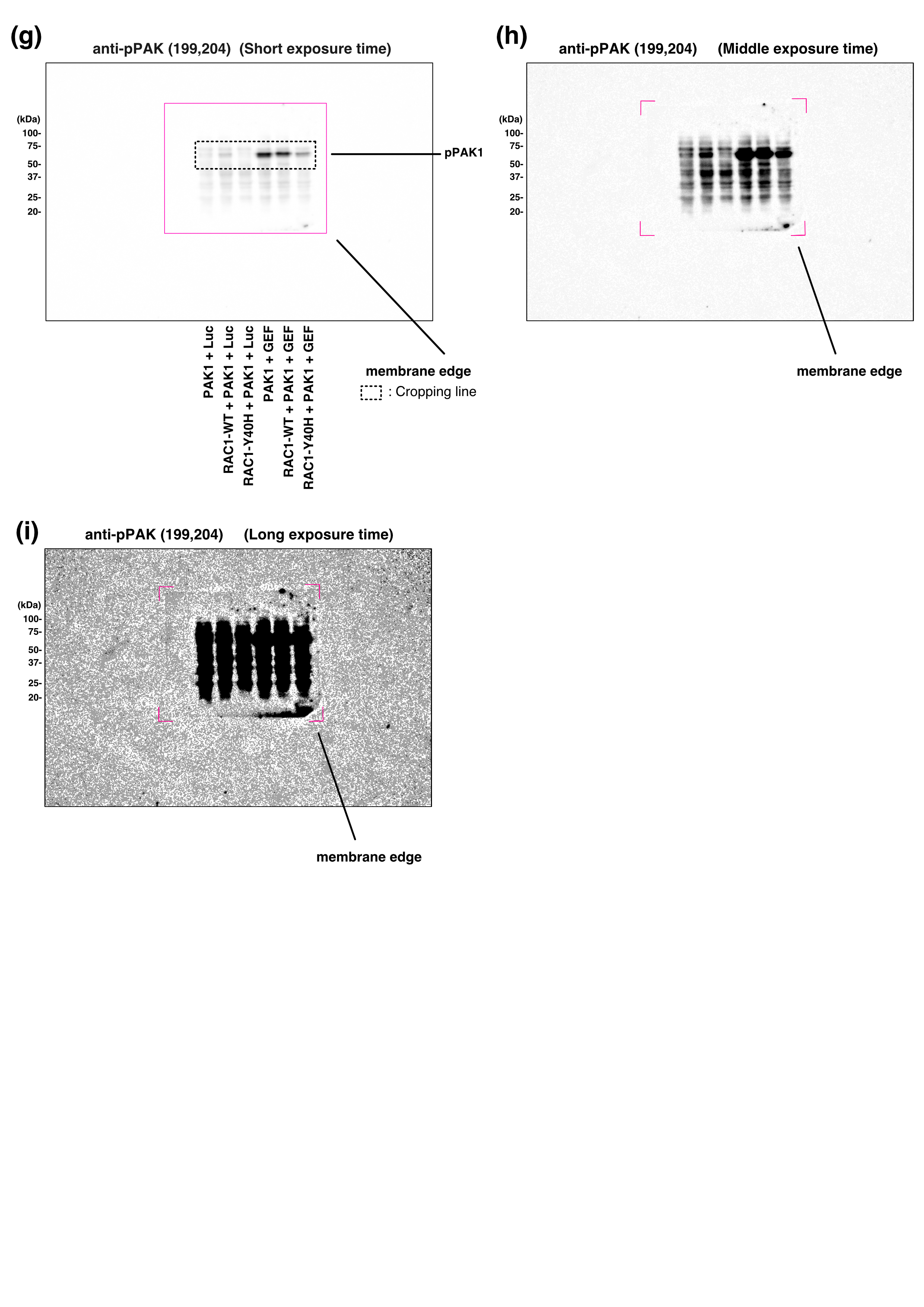


(a-c) Uncropped blot images for Fig. 4a were provided. The same membrane images were original (a), moderately processed (b), and significantly processed by photoshop (c) to clearly show the edge of membrane. Positions of RAC1 proteins, Trio-D1 (GEF), and control luciferase (Luc) were indicated in (a). The cropped regions in (a) were used for the upper and lower panels of Fig. 4a. Very low background signal made the membrane edges invisible. (d-f) Uncropped blot images for the top and middle panels in Fig. 4c were provided. The same membrane images were original (d), moderately processed (e) and significantly processed by photoshop (f). Positions of RAC1 proteins, GEF, PAK1, and Luc were indicated in (e). The cropped regions in (e) were used for the top and middle panels of Fig. 4c. (g-i) Uncropped blot images for the bottom panel in Fig. 4c were provided. The same membrane images were original (g), moderately processed (h) and significantly processed by photoshop (i). The cropped region in (g) was used for the bottom panel of Fig. 4c. The membrane was cut prior to immunoblotting. The position of phosphorylated PAK1 (pPAK1) was indicated in (a). (a-i) The membrane edges are shown by magenta lines in the short exposure images. Corners of the blots of the meddle and long exposure images were also marked.

**Fig. S3 Scheme for the molecular mechanism of the p.Tyr40His variant**

**
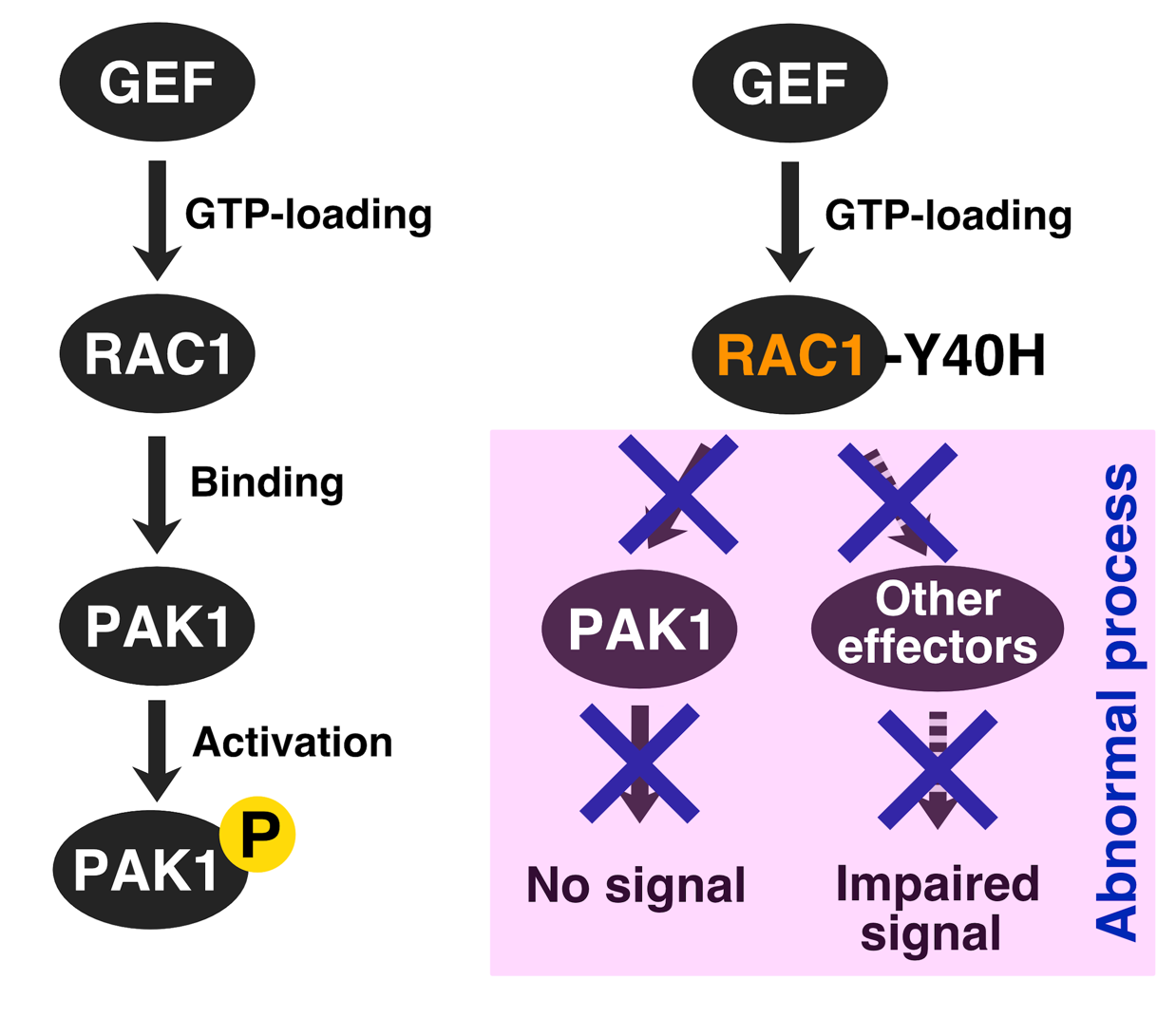
**

The RAC1-p.Tyr40His variant exhibits biochemical properties comparable to wild-type RAC1. The variant, however, does not interact with or activate the downstream effector, PAK1, even in the GTP-bound active state. This variant is also possible to impair functions of yet unidentified effectors, leading to disruption of their signaling pathways.
